# Chicken as Reservoir for Extraintestinal Pathogenic *Escherichia coli* in Humans, Canada

**DOI:** 10.3201/eid1803.111099

**Published:** 2012-03

**Authors:** Catherine Racicot Bergeron, Catharine Prussing, Patrick Boerlin, Danielle Daignault, Lucie Dutil, Richard J. Reid-Smith, George G. Zhanel, Amee R. Manges

**Affiliations:** McGill University, Montréal, Québec, Canada (C. Racicot Bergeron, C. Prussing, A.R. Manges);; University of Guelph, Guelph, Ontario, Canada (P. Boerlin, R.J. Reid-Smith);; Public Health Agency of Canada, Saint-Hyacinthe, Québec, Canada (D. Daignault, L. Dutil);; Public Health Agency of Canada, Guelph (R.J. Reid-Smith);; University of Manitoba, Winnipeg, Manitoba, Canada (G.G. Zhanel)

**Keywords:** Escherichia coli, molecular epidemiology, urinary tract infections, extraintestinal infections, retail meat, foodborne transmission, food reservoir, food animal reservoir, bacteria, Canada, antimicrobial resistance, sequence types, chicken

## Abstract

We previously described how retail meat, particularly chicken, might be a reservoir for extraintestinal pathogenic *Escherichia coli* (ExPEC) causing urinary tract infections (UTIs) in humans. To rule out retail beef and pork as potential reservoirs, we tested 320 additional *E. coli* isolates from these meats. Isolates from beef and pork were significantly less likely than those from chicken to be genetically related to isolates from humans with UTIs. We then tested whether the reservoir for ExPEC in humans could be food animals themselves by comparing geographically and temporally matched *E. coli* isolates from 475 humans with UTIs and from cecal contents of 349 slaughtered animals. We found genetic similarities between *E. coli* from animals in abattoirs, principally chickens, and ExPEC causing UTIs in humans. ExPEC transmission from food animals could be responsible for human infections, and chickens are the most probable reservoir.

## Abstract 2

Urinary tract infections can be difficult and expensive to treat. Most (85%) are caused by bacteria called *E. coli*. Historically, doctors have believed that these urinary tract *E. coli* came from the patient’s own intestines. But recently, Canadian researchers discovered that not only can these *E. coli* come from outside the patient’s intestines, they can actually come from outside the patient: from food. After comparing the genetic makeup of *E. coli* from human urinary tract infections with *E. coli* from retail meat (chicken, beef, and pork), they concluded that chickens are a likely source of *E. coli* and that the infections probably come directly from the chickens themselves, not from human contamination during food processing. Therefore, prevention of *E. coli* urinary tract infections in people might need to start on chicken farms.

Extraintestinal pathogenic *Escherichia coli* (ExPEC) is the leading cause of community-acquired urinary tract infections (UTIs) in humans, accounting for >85% of UTIs (*1*). Each year, 6–8 million UTIs are diagnosed in the United States, and 130–175 million are diagnosed worldwide. Estimated direct health care costs related to uncomplicated UTIs in the United States are $1–$2 billion per year (*1**,**2*). UTIs also can lead to more severe illnesses, such as pyelonephritis, bacteremia, and sepsis (*3*). During the past decade, the emergence of drug-resistant *E. coli* has dramatically increased. As a consequence, the management of UTIs, which was previously straightforward, has become more complicated; the risks for treatment failure are higher, and the cost of UTI treatment is increasing (*4*).

In the past, extraintestinal *E. coli* infections have been described as sporadic infections caused by bacteria that originate from the host’s intestinal tract. However, ExPEC strains recently have been associated with possible outbreaks (*5*). Communitywide outbreaks have been described in south London (*E. coli* O15:K152:H1) (*6*); Copenhagen (*E. coli* O78:H10) (*7*); Calgary, Alberta, Canada (extended-spectrum β-lactamase–producing *E. coli*) (*8*); and California, USA (trimethoprim/sulfamethoxazole–resistant *E. coli*) (*9*). These outbreaks suggest that ExPEC can be spread to the intestinal tracts of persons in the community by a common source or vehicle.

We recently described the results of a study that characterized the genetic similarities between *E. coli* isolates recovered from retail meat, particularly chicken, and ExPEC in humans causing community-acquired UTIs (*10*). That study oversampled isolates from retail chicken because evidence suggested that chicken was likely to be the primary reservoir of ExPEC in humans (*11**–**16*). To exclude the possibility that isolates from other retail meat sources (beef and pork) might also be genetically related to UTI isolates from humans, we first aimed to characterize additional *E. coli* isolates recovered from retail beef and pork sources. These new isolates from retail meat were added to the preexisting collection of retail meat isolates and compared with the same UTI isolates from humans. Second, we aimed to determine whether transmission was primarily human to human through food or whether an animal source was involved. In the case of human-to-human transmission through food, *E. coli* strains from humans would be introduced during the meat preparation process by food handlers. In the case of an animal source, the *E. coli* would derive from the cecal content of the animal itself, and contamination would occur during the slaughtering process. On the basis of previous findings, we hypothesized that a food animal reservoir exits for ExPEC that cause UTIs in humans and that chicken is the primary source. To evaluate this hypothesis, we analyzed isolates from animals entering the food chain. *E. coli* isolates recovered from the cecal contents of slaughtered food animals (beef cattle, chickens, and pigs) were compared with the preexisting geographically and temporally matched collection of isolates from humans with UTIs.

## Methods

### Study Design

A total of 1,561 geographically and temporally matched *E. coli* isolates from animals and from humans with UTIs were used for the different comparisons (Table 1).The study target area was primarily the province of Québec, Canada; however, other regions were included as described below. The study period was 2005–2007. The McGill University Institutional Review Board approved the study protocol (A01-M04-05A).

**Table 1:** Composition of closely related *Escherichia coli* clonal groups from humans and retail meat or abattoir source isolates, Canada, 2005–2007*

Source	No. (%) isolates										
	Year					Geographic area					Total
	2005	2006	2007	2008		QC	ON	BC/AB	SK/MB	Maritimes	
UTI in humans											
All	102 (21)	174 (37)	137 (29)	62 (13)		379 (80)	37 (8)	23 (5)	24 (5)	12 (3)	475 (100)
Manges collection†	102 (21)	174 (37)	75 (16)	0		351 (74)	0	0	0	0	351 (74)
Zhanel collection‡	0	0	62 (13)	62 (13)		28 (6)	37 (8)	23 (5)	24 (5)	12 (3)	124 (26)
Retail meat											
All	275 (37)	243 (33)	219 (30)	0		521 (71)	202 (27)	4 (1)	10 (1)	0	737 (100)
Beef	84 (11)	72 (10)	86 (12)	0		210 (28)	32 (4)	0	0	0	242 (33)
Chicken	107 (15)	101 (14)	45 (6)	0		141 (19)	99 (13)	3 (0.4)	10 (1)	0	253 (34)
Pork	84 (11)	70 (9)	88 (12)	0		170 (23)	71 (10)	1 (0.1)	0	0	242 (33)
Abattoir											
All	133 (38)	101 (29)	115 (33)	0		107 (31)	146 (42)	48 (14)	30 (9)	4 (1)	349 (100)
Beef	18 (5)	22 (6)	20 (6)	0		11 (3)	23 (7)	17 (5)	8 (2)	1 (0.3)	60 (17)
Chicken§	89 (26)	60 (17)	80 (23)	0		75 (21)	104 (30)	27 (8)	10 (3)	2 (1)	229 (66)
Pig¶	26 (7)	19 (5)	15 (4)	0		21 (6)	19 (5)	4 (1)	12 (3)	1 (0.3)	60 (17)
Total	510 (33)	518 (33)	471 (30)	62 (4)		1,007 (65)	385 (25)	75 (5)	64 (4)	16 (1)	1,561 (100)

*QC, Québec; ON, Ontario; BC, British Columbia; AB, Alberta; SK, Saskatchewan; MB, Manitoba; Maritimes, New Brunswick/Nova Scotia/Prince Edward Island; UTI, urinary tract infection. †351 *E. coli* isolates recovered from humans with UTIs in Montréal during June 2005–May 2007. ‡124 *E. coli* isolates from humans with community- or hospital-acquired UTIs from sources throughout Canada during 2007–2008. §The geographic area was unknown for 11 isolates from chicken. ¶The geographic area was unknown for 3 isolates from pigs.

### Sampling of *E. coli* Isolates from Humans with UTI, Montreal, Québec

The 351 *E. coli* isolates recovered from humans with UTIs were collected in Montréal during June 2005–May 2007 (*17*). Women 18–45 years of age who had a suspected UTI were recruited at the McGill University Student Health Services and the Centre Local de Services Communautaires Métro Guy. UTI was defined as >2 symptoms or signs, including dysuria, increased urinary frequency or urgency, pyuria, hematuria, and >10^2^ CFU of *E. coli* per mL of clean-catch urine (*18*). Specimen culture and bacterial identification have been described (*17*). One random isolate from each urine sample was selected. In case of UTI recurrence, only the isolate from the first UTI episode was included. The collection was assembled as follows. A random set of 116 fully susceptible isolates was selected. A random set of 170 isolates resistant to >1 antimicrobial agents was assembled; in addition, specific groups of antimicrobial-resistant *E. coli* were included. These *E. coli* strains have been closely associated with possible outbreaks of extraintestinal infections (*6**–**9**,**19**–**21*). In particular, cephalosporin-resistant *E. coli* frequently has been observed in UTI outbreaks and in poultry products (*8**,**13**,**16**,**19**–**21*). Hence, all 19 cephalothin-resistant *E. coli* isolates were included. We included 46 representative members of *E. coli* clonal groups that were known to cause clusters of UTI among unrelated women on the basis of the hypothesis that they would be more likely to be related to food sources. This collection of 351 UTI-associated *E. coli* isolates is referred to as the Manges collection.

### Sampling of *E. coli* Isolates from Humans with UTIs, Canada

We added 124 isolates from humans from sources across Canada to increase the diversity of the collection of *E. coli* isolates causing UTIs beyond those in Québec alone. These samples were collected from patients with community- and hospital-acquired UTIs during 2007–2008. This collection, provided by 1 of the authors (G.G.Z.), is referred to as the Zhanel collection.

### Sampling of *E. coli* from Retail Meat

We systematically selected and evaluated additional isolates from retail beef and pork. The retail meat collection totaled 737 isolates from beef (242), chicken (253), and pork (242) (*10*). These isolates were collected in Montréal, areas of Québec outside Montréal, parts of Ontario, and other areas of Canada during 2005–2007. All of these isolates originated from the collection of the Canadian Integrated Program for Antimicrobial Resistance Surveillance (CIPARS). Because antimicrobial resistance has been associated with ExPEC clonal groups and outbreaks of UTIs (*6**–**9**,**17**,**22*), we oversampled antimicrobial-resistant isolates from retail meat; 60% of sampled isolates were resistant to >1 antimicrobial drugs, and 40% were fully susceptible. However, resistance in retail beef was fairly low; therefore the proportion of resistant *E. coli* from retail beef was only 48%.

### Sampling of *E. coli* from Food Animals in Abattoirs

From the CIPARS collection, we selected 349 *E. coli* isolates from animals in abattoirs. These bacteria were isolated from the cecal contents of slaughtered food animals (*23*). Because the primary hypothesis concerned a chicken reservoir and we already had demonstrated that isolates from humans are less likely to be related to isolates from beef and pork, we included isolates in the following proportions: 20% beef cattle, 60% chickens, and 20% pigs. We chose 299 isolates from 2005–2007 as follows. Isolates from chickens were selected from abattoirs in Québec and Ontario because there are poultry abattoirs operating in Québec and Ontario and because *E. coli* from humans with UTIs was recovered primarily from women in Québec. In contrast, beef cattle and pig abattoirs are fewer and are located across Canada. Therefore, isolates were selected on the basis of the annual slaughter volume rather than on location. Sampling was conducted in proportion to the susceptibility levels for each animal species within the whole CIPARS collection. However, we included all 5 nalidixic acid–resistant isolates because this agent can be used as an indicator of resistance to fluoroquinolones (*24*).

Furthermore, because the study focused on chicken, we included a random sample of 50 additional isolates from chickens in abattoirs in other Canadian provinces. They were also selected from the CIPARS collection during the same period (2005–2007).

### Antimicrobial Susceptibility Testing

We performed antimicrobial susceptibility testing on all *E. coli* isolates, except those from the Zhanel collection. Antimicrobial susceptibility screening using a panel of 15 agents (amikacin, amoxicillin/clavulanic acid, ampicillin, cefoxitin, ceftiofur, ceftriaxone, chloramphenicol, ciprofloxacin, gentamicin, kanamycin, nalidixic acid, streptomycin, sulfisoxazole, tetracycline, and trimethoprim/sulfamethoxazole) was conducted by the Public Health Agency of Canada, Laboratory for Foodborne Zoonoses. The protocol of the broth microdilution method used was fully described in the 2007 CIPARS report (*23*). Intermediate resistance for all isolates was classified as susceptible. The antimicrobial resistance patterns are provided for informational purposes only because isolates were sampled in part according to their antimicrobial resistance phenotype. Thus, the patterns observed do not reflect the prevalence of antimicrobial resistance for any of the sources.

### Clonal Group Definition and Typing

Isolates were typed by multilocus variable number tandem repeat analysis (MLVA) (*25*) and enterobacterial repetitive intergenic consensus sequence 2 (ERIC2) PCR fingerprinting (*26*) in our laboratory and the McGill University and Genome Québec Innovation Center as described. A clonal group was defined as >2 *E. coli* isolates from human and animal sources that shared the same MLVA profile and ERIC2 PCR fingerprint. All clonal group members were further typed by phylotyping and multilocus sequence typing (MLST). Then, according to the results obtained, related isolates were selected for O:H serotyping and pulsed-field gel electrophoresis (PFGE).

Phylotyping (*27*) and MLST (*28*) (http://mlst.ucc.ie/mlst/dbs/Ecoli) were performed in our laboratory and in the McGill University and Genome Québec Innovation Center as described. Allelic profile and sequence type (ST) were assigned according to the scheme at this website. O (somatic) and H (flagellar) antigens were serotyped for clonal group isolates that shared the same phylogenetic group and MLST profile; serotyping was performed by the Public Health Agency of Canada, Laboratory for Foodborne Zoonoses, according to established protocols. Isolates that did not react with O antiserum were classified as nontypeable. PFGE of *Xba*I*-*digested DNA was conducted in our laboratory by using the protocol for molecular subtyping of *E. coli* O157:H7 developed by the Centers for Disease Control and Prevention (*29*). Clonal group members that exhibited the same phylotype and MLST profile were tested. Gel fingerprints were visually compared, and strain relatedness was classified according to the Tenover criteria (*30*).

### Statistical Analyses

Differences in proportions were assessed by use of the χ^2^ test. Statistical significance was defined as a p value <0.05. All analyses were conducted using Stata version 9.0 (StataCorp LP, College Station, TX, USA).

## Results

### Isolates from Retail Meat

We identified 15 clonal groups, comprising 63 isolates. The 15 groups contained 22 isolates from humans with UTIs and 41 isolates from retail meat. Of the 41 isolates from retail meat, 6 (15%) were from retail beef, 29 (71%) from retail chicken, and 6 (15%) from retail pork. Considering the sampling proportions (66% beef and pork [484/737] and 34% chicken [253/737]), the fraction of isolates from beef and pork related to isolates from humans with UTIs was significantly lower than expected on the basis of the sampling fraction (29% observed vs. 66% expected; p<0.001, χ^2^ test). Isolates from chicken were represented in greater numbers among clonal groups. All clonal group members had the same phylotype. According to MLST, 2 of these clonal groups (groups 1 and 3) contained isolates from newly sampled retail beef and pork (EC01DT07-0827-01 and EC01DT06-1559-01) and isolates from humans with UTIs that shared the same STs. These isolates were further typed (Table 2): *Xba*I PFGE patterns differed by >7 bands within the clonal groups associated with retail meat.

**Table 2:** Composition of closely related clonal groups containing extraintestinal pathogenic *Escherichia coli* from humans and retail meat or abattoir source isolates, Canada, 2005–2007*

Clonal group/strain	Type of sample	Isolate source	Year	Location	Phylotype	ST	Serotype	Antimicrobial drug susceptibility or resistance†
1								
MSHS 1014A	Clinical	CA-UTI	2007	QC	D	117	O114:H4	Susceptible
EC01DT05-0789-01	Retail meat	Chicken	2005	ON	D	117	O114:H4	Susceptible
EC01AB06-0065-01	Abattoir	Chicken	2006	QC	D	117	O2:H4	GEN, SIX, TET
MSHS 133	Clinical	CA-UTI	2005	QC	D	117	O24:NM	TET
EC01DT07-1090-01	Retail meat	Chicken	2007	QC	D	117	O24:H4	GEN, SIX, TET
EC01DT07-1050-01	Retail meat	Chicken	2007	ON	D	117	O45:H4	Susceptible
EC01DT07-0956-01	Retail meat	Chicken	2007	SK	D	117	O53:H4	AMP, NAL, STR, SIX, TET, TMP/SXT
EC01AB07-0840-01	Abattoir	Chicken	2007	ON	D	117	O53:NM	Susceptible
EC01AB07-0615-01	Abattoir	Chicken	2007	ON	D	117	O102:H4	AMC, AMP, FOX, TIO STR, SIX, TET
EC01AB05-1250-01	Abattoir	Chicken	2005	ON	D	117	O103:H4	AMC, AMP
EC01DT06-1887-01	Retail meat	Chicken	2006	QC	D	117	O143:H4	Susceptible
EC01AB06-1131-01	Abattoir	Pig	2006	QC	D	117	O143:H4	SIX, TET
EC01AB07-0695-01	Abattoir	Chicken	2007	ON	D	117	O149:H4	STR, SIX, TET
EC01DT05-1700-01	Retail meat	Chicken	2005	QC	D	117	O160:H4	Susceptible
EC01AB07-0105-01	Abattoir	Chicken	2007	ON	D	117	O180:H4	Susceptible
EC01AB07-0425-01	Abattoir	Chicken	2007	BC	D	117	O180:H4	KAN, STR, SIX, TET
EC01DT07-0827-01	Retail meat	Pork	2007	ON	D	117	ONT:H4	STR, SIX
EC01AB05-0695-01	Abattoir	Chicken	2005	ON	D	117	ONT:H4	GEN, SIX,
EC01DT05-0224-01	Retail meat	Chicken	2005	ON	D	117	OX182:NM	Susceptible
2								
MSHS 624	Clinical	CA-UTI	2006	QC	A	746	O20:H4	Susceptible
EC01AB07-1301-01	Abattoir	Pig	2007	ON	A	746	O20:NM	AMP, CHL, SIX, TET
EC01AB05-0990-01	Abattoir	Chicken	2005	ON	A	746	O87:NM	AMC, AMP, FOX, TIO, CHL, STR, SIX, TET
EC01DT06-0006-01	Retail meat	chicken	2006	QC	A	746	O33:NM	Susceptible
EC01AB05-0091-01	Abattoir	Pig	2005	ON	A	10		Susceptible
MSHS 254	Clinical	CA-UTI	2005	QC	A	None		CEF, TET
48-75641	Clinical	CA-UTI	2007	Maritimes	A	None		
3								
EC01AB05-0765-01	Abattoir	Chicken	2005	ON	A	10	O16:H48	Susceptible
EC01AB07-0005-01	Abattoir	Chicken	2007	ON	A	10	O16:H48	STR, SIX, TET
EC01AB07-1330-01	Abattoir	Chicken	2007	BC	A	10	O16:H48	Susceptible
MSHS 233	Clinical	CA-UTI	2005	QC	A	10	O9:H32	AMP, TET
MSHS 825A	Clinical	CA-UTI	2006	QC	A	10	O15:NM	AMP, CHL, STR, SIX, TET, TMP/SXT
EC01DT06-1559-01	Retail meat	Pork	2006	ON	A	10	O42:H37	Susceptible
MSHS 892	Clinical	CA-UTI	2006	QC	A	10	O101:NM	CIP, KAN, NAL, STR, TET
120-79443	Clinical	HA-UTI	2008	Maritimes	A	10	O101:H9	
EC01DT05-1925-01	Retail meat	Chicken	2005	QC	A	10	O106:H4	Susceptible
EC01DT05-0408-01	Retail meat	Chicken	2005	QC	A	10	O153:NM	AMC, AMP, FOX, TIO STR, SIX, TET, TMP/SXT
EC01DT07-1162-01	Retail meat	Chicken	2007	ON	A	10	OX182:NM	AMC, AMP, FOX, TIO, TET
EC01DT07-0491-01	Retail meat	Chicken	2007	QC	A	10	OX184:H4	NAL, TET
EC01AB06-0855-01	Abattoir	Chicken	2006		A	548		AMP, KAN, STR, SIX, TET
51-77552	Clinical	CA-UTI	2008	SK/MB	A	None		
EC01DT06-1546-01	Retail meat	Chicken	2006	QC	A	None		AMP, GEN, SIX, TET
EC01AB05-0320-01	Abattoir	Chicken	2005	QC	A	None		Susceptible
EC01AB06-0119-01	Abattoir	Pig	2006	SK	A	None		KAN, TET
EC01AB06-1005-01	Abattoir	Chicken	2006	ON	A	None		Susceptible
EC01AB07-0566-01	Abattoir	Pig	2007	SK	A	None		STR, SIX

*ST, sequence type; MSHS, McGill University Student Health Services; CA-UTI, community-acquired urinary tract infection; QC, Québec; ON, Ontario; AB, Alberta; GEN, gentamicin; SIX, sulfisoxazole; TET, tetracycline; NM, nonmotile; SK, Saskatchewan; AMP, ampicillin; NAL, nalidixic acid; TMP/SXT, trimethoprim/sulfamethoxazole; AMC, amoxicillin/clavulanic acid; FOX, cefoxitin; TIO ceftiofur; STR, streptomycin; CEF, cephalothin; Maritimes, New Brunswick/Nova Scotia/Prince Edward Island; BC, British Columbia; ONT, did not react with O antiserum; KAN, kanamycin; CHL, chloramphenicol; CIP, ciprofloxacin; HA-UTI, hospital-acquired urinary tract infection; MB, Manitoba; blank cells indicate isolate not tested. †Resistance to specific antimicrobial drugs as indicated.

### Isolates Collected from Abattoirs

We identified 8 clonal groups containing 46 isolates from humans and from food animals at abattoirs. These clonal groups comprised 17 isolates from humans with UTIs and 29 from abattoir animals (1 [3%] beef cattle, 23 [79%] chickens, 5 [17%] pigs). The proportion of chicken was higher than expected with 79% observed versus 60% expected, in accordance with the 60% sampling fraction (p = 0.034, χ^2^ test). The 3 clonal groups including isolates that were further characterized, and thus more closely related, are described below (Table 2). According to the Tenover criteria (*30*), PFGE patterns of all animal strains differed from those of the human strains.

Abattoir clonal group 1 contained 11 isolates (2 from humans, 8 from chickens, 1 from a pig). All belonged to the same phylogenetic group (D) and showed the same sequence type (ST117). Two isolates from chickens (EC01AB07-0105-01 and EC01AB07-0425-01) had the same serotype (O180:H4), and the rest of the isolates had unique serotypes.

Abattoir clonal group 2 included 6 isolates (3 from humans, 1 from a chicken, 2 from pigs). They all belonged to phylogenetic group A but showed 4 different MLST profiles. Among them, ST746 was shared by 3 isolates (1 each from a human, chicken, and pig). However, they did not show the same O:H serotype.

Abattoir clonal group 3 contained 13 isolates (5 from humans, 6 from chickens, 2 from pigs), which all belonged to phylogenetic group A. The MLST profile ST10 was shared by 7 isolates (4 from humans, 3 from chickens). The 6 other isolates displayed different MLST profiles. Among the isolates exhibiting ST10, 3 from chickens (EC01AB05-0765-01, EC01AB07-0005-01, and EC01AB07-1330-01) showed the same serotype (O16:H48).

## Discussion

Our first goal was to exclude retail beef and pork as a probable food source of *E. coli* causing UTIs. Our previous study (*10*), in which the sampling proportions for beef, chicken, and pork were not the same, clonal groups identified included 17% isolates from beef and pork and 83% from chicken (p = 0.03). In the current investigation, where the sampling proportions from retail meat were the same, 12 (29%) of isolates belonging to clonal groups were from beef and pork and 29 (71%) were isolated from chicken (p<0.001). Retail beef and pork isolates are much less likely than retail chicken isolates to be clonally related to isolates from humans with UTIs.

Our second goal was to determine whether the reservoir for ExPEC in humans causing community-acquired UTI was food animals, particularly chickens. The initial screening methods (MLVA and ERIC2) demonstrated that human samples and cecal samples from food animals in abattoirs can belong to the same clonal groups. Moreover, within certain abattoir clonal groups, isolates showed the same phylogenetic group and MLST sequence types, indicating that they may have originated from a recent common ancestor. The 3 major clonal groups with the highest level of similarity (groups 1, 2, and 3) included isolates from abattoir and retail meat (*10*), which suggests that food animals may serve as a reservoir for ExPEC in humans.

The 2 most common STs (ST10 and ST117), belonging to phylogenetic groups A and D, respectively, have already been reported from human and animal sources (*11**,**31**–**33*). Although phylogenetic group A is typically associated with commensal *E. coli* (*3*), most human and animal isolates from the abattoir clonal groups belonged to this phylotype. Moreno et al. (*34*) and Ewers et al. (*35*) reported data suggesting that isolates from phylogenetic group A could be responsible for extraintestinal infections. Phylogenetic group D, which has frequently been associated with ExPEC in humans (*3*), was observed in 31% of the isolates from abattoir clonal groups. The lack of isolates from phylogenetic group B2 was unexpected because extraintestinal pathogenic strains often belong to this group (*3*). Studies from Jakobsen et al. have identified phylogroup B2 isolates from meat and animal sources, which demonstrates that B2 exists in the food animal reservoir (*36**,**37*). Our results may be explained by the fact that the isolates collected from abattoirs are more likely to be generic or commensal *E. coli* rather than typical ExPEC because they were collected from the cecal contents of healthy animals. The lack of phylogenetic group B2 isolates also could be explained by sampling variability or our selection method (based on MLVA and ERIC2 PCR genotyping first, followed by phylotyping). Phylogenetic group A and D were predominant among the isolates collected from abattoirs, which is consistent with results obtained by Jakobsen et al. (*38*) and Cortés et al. (*11*).

Although we oversampled isolates from abattoir chickens (60%), a significantly higher proportion of the isolates collected from abattoirs (79%; p *=* 0.034) included in the clonal groups were from chickens than from beef cattle or pigs; this proportion was higher than expected. This study confirms our hypothesis that chickens are a likely reservoir for ExPEC in humans. However, epidemiologic data, such as diet or other exposures, were not available for the humans with UTIs. This information could have been used to search for other potential routes of transmission (e.g., travel, water sources) and to strengthen the connection between poultry consumption and UTI.

We observed more heterogeneity in the PFGE results than in results from the other typing methods. PFGE is the standard for genotyping *E. coli* in the context of outbreaks, but it is generally not useful for establishing relationships between isolates from greater distances and over longer periods (*12*). MLST results may be a more relevant as housekeeping genes evolve more slowly and are more appropriate for examining questions related to global or regional epidemiology (*39**,**40*).

This study was strengthened by use of an ecologic design in which all isolates were systematically and purposively selected over the same period of time and geographic area (*17**,**23*), rather than sampling haphazardly by using existing clinical laboratory collections. The results suggest that potential ExPEC transmission from food animal sources is likely to be implicated in human infections and that chicken is a major reservoir. The possibility that ExPEC causing UTIs and other extraintestinal infections in humans could originate from a food animal reservoir raises public health concern. New interventions may be needed to reduce the level of food contamination and risk for transmission.
